# Current treatments against mucormycosis and future directions

**DOI:** 10.1371/journal.ppat.1010858

**Published:** 2022-10-13

**Authors:** Courtney Smith, Soo Chan Lee

**Affiliations:** South Texas Center for Emerging Infectious Diseases (STCEID), Department of Molecular Microbiology and Immunology, The University of Texas at San Antonio, San Antonio, Texas, United States of America; Vallabhbhai Patel Chest Institute, INDIA

## Abstract

Mucormycosis (previously called zygomycosis) is a serious but rare fungal infection caused by a group of fungi belonging to the order Mucorales. These molds exist throughout the environment and generally do not cause serious problems in humans. Mucormycosis mainly affects individuals who are immunocompromised. The clinical manifestations of mucormycosis are wide-ranging; they include sinusitis (pansinusitis, rhino-orbital, or rhino-cerebral) as well as cutaneous, gastrointestinal, pulmonary, and disseminate infections. Many uncertainties remain regarding how to control these infections despite the recent addition of triazoles to the antifungal arsenal for treating this infection. Currently, lipid formulations of amphotericin B have become the standard treatment for mucormycosis due to their efficiency. Moreover, a growing body of data supports the need for surgical excision of infected and/or necrosed tissue whenever practical. In this mini review, the current status of treatment options for mucormycosis and recent studies of novel therapeutic options will be presented.

## Introduction

Mucormycosis is a fungal infection caused by fungi belonging to the order Mucorales, which include *Mucor* spp., *Rhizopus* spp., *Rhizomucor*, *Lichtheimia* spp., and others. These organisms are ubiquitous in the environment, so most individuals are exposed to them on a daily basis [1]. Mucormycosis is an opportunistic fungal infection; therefore, individuals with lowered immune systems are at higher risk of developing an infection. Recent data indicate a significant increase in mucormycosis as a result of the growing population of immunocompromised patients with conditions such as diabetes, hematologic malignancies, hematopoietic stem cell/solid organ transplant, or trauma [2]. Additionally, there was an outbreak of mucormycosis among individuals in India with Coronavirus Disease 2019 (COVID-19), specifically in patients with uncontrolled diabetes [3]. Hypoxemia related to COVID-19 and overuse of glucocorticoid are also involved in this massive outbreak of mucormycosis. A high level of iron in COVID-19 patients due to hyperferritinaemia may have increased the probability of mucormycosis [4]. COVID-19-related mucormycosis cases later were observed worldwide [5]. The clinical presentation of mucormycosis varies. The most dramatic clinical manifestation is in patients with diabetes who have ketoacidosis; these patients tend to have rhino-cerebral instead of pulmonary mucormycosis [6]. Even though the number of cases of Mucorales infection among the immunocompromised population is few compared to candidiasis, cryptococcosis, or aspergillosis, the consequences are severe due to its high mortality rate. Also, mucormycosis often is misdiagnosed and is in many cases diagnosed only postmortem.

Addressing the complete spectrum of mucormycosis is beyond the scope of this review, and the focus is on the status of the management and treatment of mucormycosis. The principles behind treating and controlling mucormycosis are multifactorial and are based on utilizing several interventions concurrently. These interventions include but are not limited to early clinical diagnosis, effective mono- or combination antifungal therapy, surgical debridement of necrotic lesions, and control of underlying medical conditions (e.g., diabetes) [7]. Early diagnosis and treatment are essential to increase the chance of survival; no treatment is not an option because untreated mucormycosis is almost inevitably fatal.

## What are the currently available antifungal drugs?

### Amphotericin B and its lipid formulations

**Fig 1:**
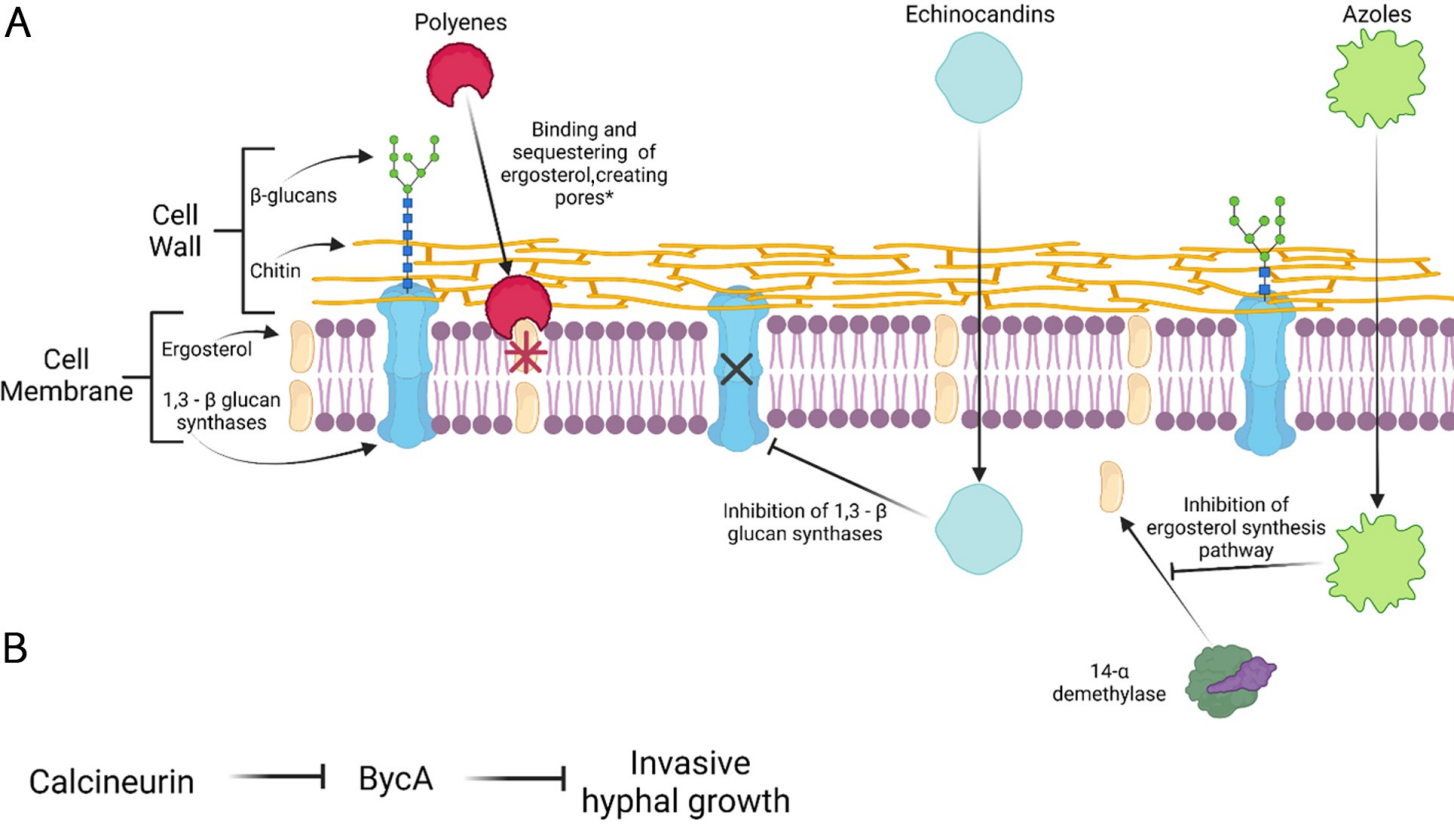
Diagram representing the current drug targets and mechanism of action for mucormycosis. (A) Polyenes, such as AMB, targets ergosterol and functions by binding and sequestering the ergosterol resulting in cell membrane instability and pore formation. Echinocandins display fungistatic activity by entering the fungal cell and noncompetitively inhibits 1,3 –β glucan synthase, an enzyme that is necessary for the assembly of an essential component of the cell wall of several fungi. Azoles block the synthesis of cell membrane-stabilizing ergosterol via the inhibition of lanosterol 14- α—demethylase enzyme. (B) Calcineurin is a regulator of *Mucor* morphology; active calcineurin promotes hyphal growth. This regulation is maintained by suppressing the expression of the *bycA* gene. *Additional models for the mechanism of action for AMB have been proposed; one is the sterol sponge model where AMB is laterally adsorbed onto the membrane surface of the fungus. This figure was created with BioRender.com.

Amphotericin B (AMB) and its lipid formulations (including liposomal preparation, a lipid complex, and a colloidal dispersion) target ergosterol in the fungal walls, resulting in cell wall destabilization, and are the first FDA-approved drug for treating mucormycosis infections (Fig 1A) [8]. AMB lipid formulations (liposomal AMB, LAMB, and the AMB lipid complex, ABLC) have improved therapeutic indexes when compared to conventional AMB. However, it has been noted that in vitro AMB activity against Mucorales varies greatly [9]. Based on recent in vivo and in vitro studies, it is proposed that to treat mucormycosis effectively, one must consider the availability of surgical debridement and antifungal treatments. The optimal dosage of these drugs when treating mucormycosis is dependent on the disease areas and the patient’s condition [10]. When the infection involves the brain or occurs in solid organ transplant recipients, a high dose of LAMB and ABLC (10 mg/kg/day) is considered therapeutic [11]. Depending on the response to the first line of treatment, the continuation of LAMB/ABLC treatment at a high dose is recommended when the disease is stable or there is only a partial response to the drugs. On the other hand, in the case of progressive disease, treatment with LAMB or ABLC in combination with posaconazole is recommended. If toxicity is observed in the patient after the first line of treatment, low-dose LAMB or ABLC (5 mg/kg/day) treatment is recommended. However, these drugs are associated with increased nephrotoxicity and electrolyte imbalances [8]. If this occurs, isavuconazole and posaconazole are recommended [10]. LAMB or ABLC (5 mg/kg/day) treatment is also recommended in COVID-19-associated mucormycosis (CAM). However, AMB deoxycholate can also be recommenced for CAM when AMB lipid formulations are not available, which is common in developing countries [12].

### Triazoles: Posaconazole and isavuconazole

Triazoles act by inhibiting the ergosterol synthesis pathway (Fig 1A). Both posaconazole and isavuconazole are members of the triazole drug class; these drugs are relatively new to the group of drugs for mucormycosis, and both exhibit higher activity against Mucorales in vitro than other triazoles [13,14]. On the other hand, other triazole antifungals, including fluconazole, itraconazole, and voriconazole, exhibit minimal to no activity against Mucorales. Posaconazole has varying activity against Mucorales, and it appears that the drug’s effectiveness is species dependent [15]. This drug is generally well tolerated except for minor gastrointestinal side effects due to oral administration [16]. However, clinical data have previously demonstrated the drug’s limited effectiveness for mucormycosis, which may lead to poor efficacy of the drug [17,18]. Early data suggest that posaconazole may be a salvage therapy option for patients who are nonresponsive to other treatments. Isavuconazole is also a broad-spectrum triazole that has recently been approved to treat mucormycosis when AMB is not sufficient or as a first-line treatment option. Compared to other triazoles, this drug has more advantages, which include less drug–drug interactions, decreased toxicity, and availability in intravenous and oral formulations. Like posaconazole, isavuconazole activity against Mucorales is species dependent, and it can be used as a salvage therapy [19].

A recent guideline summarizes the usage of posaconazole and isavuconazole to treat mucormycosis [10]. When the patient has preexisting kidney complications, or in some cases as a first-line treatment [20], intravenous administration of isavuconazole or posaconasole is recommended. This is also the case for CAM [12]. In some cases, when neither posaconazole nor isavuconazole is available, itraconazole is recommended for CAM. After assessment of the response to the first-line treatment, continuation of first-line azole treatment is recommended if the disease is stable or progressive or if there is only a partial response to the treatment.

### Combination therapy

A combination therapeutic approach is common for the severely immunocompromised population. This methodology has clinical benefits that include the synergistic effects of the drugs and a broader coverage against pathogens compared to monotherapy [21]. AMB is commonly paired with other antifungals such as echinocandins (e.g., caspofungin and micafungin). The echinocandins’ mode of action is inhibition of β-(1,3)-D-glucan synthase; this is a key enzyme for maintaining the fungal cell wall (Fig 1A). One retrospective study shows evidence of synergism between polyenes (e.g., AMB) and echinocandins. This study was conducted among diabetic patients with mucormycosis, and patients who received the combination treatment had superior outcomes compared to those who received polyene monotherapy [22].

Other combination drug therapy consists of AMB and triazoles, although the data on this combination are contradictory. In vitro studies have demonstrated a synergistic effect between polyenes and posaconazole, but in murine models, this combination shows no benefit. However, a combination treatment with LAMB and isavuconazole shows a synergistic improvement in a murine mucormycosis model [23]. This discrepancy may be due to the various Mucorales species and their different responses to antifungal drugs.

### Surgery

Necrosis and thrombosis during mucormycosis can result in poor delivery of antifungal drugs. Consequently, the elimination of affected tissue may serve as a critical form of care to completely eradicate the infection [24]. It should also be noted that outcomes of surgery are difficult to predict because of selection biases. The preferred surgical approach depends upon the patient’s status. Open surgeries are performed when the disease is extensive, whereas endoscopic surgeries are done when the disease is limited [25]. Surgical management in patients with rhino-orbito-cerebral mucormycosis has been reported to give improved results over treatment without surgery and leads to local control of the infection [26].

## What are potential future antifungal agents?

Therapeutic options are limited for mucormycosis when compared to other pathogenic infections. Currently, only two drug agents (i.e., polyenes and azoles) are used in clinical settings. It is challenging to find a distinctive drug target for Mucorales. Recent sequencing has revealed evidence of whole-genome duplication events, which might explain this pathogen’s resistance to multiple antifungal agents [27].

Presently, there are two agents under investigation: oteseconazole (VT-1161) and fosmanogepix (APX001) in clinical trials for other pathogenic fungi. Oteseconazole is an oral tetrazole and an inhibitor of fungal Cyp51; this is the same enzyme as Erg11, which encodes for lanosterol 14-α-demethylase. This enzyme is responsible for the synthesis of sterols. Therefore, the inhibition of Erg11/Cyp51 ultimately results in the cell membrane being depleted of ergosterol. Additionally, in vivo studies showed the administration of oteseconazole prolonged survival when given as a therapeutic or as a prophylaxis [28]. Fosmanogepix targets Gwt1, a protein necessary in the early steps of the conserved glycosylphosphotidyl inositol (GPI) pathway. Administration of fosmanogepix has been shown to significantly increase the survival rate and decrease fungal burden [29]. In addition, fosmanogepix exhibits higher therapeutic potential in combination with a polyene in a murine mucormycosis model [30]. A newer inhalation formulation, triazole opelconazole, exhibits activity against *Rhizopus* species [31]. These results support the potential of these drugs to serve as new antifungal agents against mucormycosis.

Calcineurin is a Ser/Thr phosphatase and regulates virulence factors in many pathogenic fungi. Calcineurin has therefore been considered a promising drug target. The phosphatase plays key roles in controlling the invasive hyphal growth in *Mucor*, in which inhibition of or mutations in calcineurin result in the blocking of hyphal growth of the fungus and the exhibition of only yeast-locked growth [32]. However, calcineurin is highly conserved in fungi and humans. Therefore, direct pharmacological inhibition of calcineurin can pose a risk of lowering host immunity as human calcineurin is required for T-cell responses [33]. It is therefore imperative to understand the downstream factors of calcineurin to develop an alternative way(s) to target calcineurin without this risk.

A recent study demonstrated that an amino acid permease (BycA) is a key downstream factor of calcineurin in *Mucor* [32]. Calcineurin negatively regulates the expression of the *bycA* genes, and without calcineurin function, the expression of the gene is elevated, resulting in the blocking of hyphal growth. It appears that up-regulation of *bycA* can recapitulate the calcineurin inhibition phenotype. Thus, any molecules that elevate the expression of *bycA* may be excellent drug candidate(s) (Fig 1B).

### Future directions/conclusions

Over the years, multiple options for mucormycosis treatments have become available. AMB, isavuconazole, and posaconazole, and the surgical debridement of necrotic tissue when necessary are the most common practices in treating mucormycosis. Existing clinical data on mucormycosis treatment are very limited and thus are not helpful for physicians. Unmet needs related to diagnosis, treatment, and education of mucormycosis lead to a poor prognosis for patients. Increasing our knowledge and understanding of these organisms is highly important in the advancement of treatments for mucormycosis.

## References

[1] Gomes MZ, Lewis RE, Kontoyiannis DP. Mucormycosis caused by unusual mucormycetes, non-Rhizopus, -Mucor, and -Lichtheimia species. Clin Microbiol Rev. 2011;24(2):411–45. Epub 2011/04/13. doi: 10.1128/CMR.00056-10 ; PubMed Central PMCID: PMC3122490. 21482731 PMC3122490

[2] Kauffman CA. Zygomycosis: Reemergence of an Old Pathogen. Clin Infect Dis. 2004;39(4):588–590. doi: 10.1086/422729 15356828

[3] Patel A, Agarwal R, Rudramurthy SM, Shevkani M, Xess I, Sharma R, et al. Multicenter Epidemiologic Study of Coronavirus Disease-Associated Mucormycosis, India. Emerg Infect Dis. 2021;27(9):2349–59. Epub 20210604. doi: 10.3201/eid2709.210934 ; PubMed Central PMCID: PMC8386807. 34087089 PMC8386807

[4] Prakash H, Skiada A, Paul RA, Chakrabarti A, Rudramurthy SM. Connecting the Dots: Interplay of Pathogenic Mechanisms between COVID-19 Disease and Mucormycosis. J Fungi (Basel). 2021;7(8). Epub 20210729. doi: 10.3390/jof7080616 ; PubMed Central PMCID: PMC8400165. 34436155 PMC8400165

[5] Hoenigl M, Seidel D, Carvalho A, Rudramurthy SM, Arastehfar A, Gangneux JP, et al. The emergence of COVID-19 associated mucormycosis: a review of cases from 18 countries. Lancet Microbe. 2022;3(7):e543–e52. Epub 20220125. doi: 10.1016/S2666-5247(21)00237-8 ; PubMed Central PMCID: PMC8789240. 35098179 PMC8789240

[6] Lanternier F, Dannaoui E, Morizot G, Elie C, Garcia-Hermoso D, Huerre M, et al. A Global Analysis of Mucormycosis in France: The RetroZygo Study (2005–2007). Clin Infect Dis. 2012;54(suppl_1):S35–S43. doi: 10.1093/cid/cir880 22247443

[7] Walsh TJ, Gamaletsou MN, McGinnis MR, Hayden RT, Kontoyiannis DP. Early clinical and laboratory diagnosis of invasive pulmonary, extrapulmonary, and disseminated mucormycosis (zygomycosis). Clin Infect Dis. 2012;54(Suppl 1):S55–60. Epub 2012/01/25. doi: 10.1093/cid/cir868 . 22247446

[8] Sipsas NV, Gamaletsou MN, Anastasopoulou A, Kontoyiannis DP. Therapy of Mucormycosis. J Fungi (Basel). 2018;4(3):90. doi: 10.3390/jof4030090 . 30065232 PMC6162664

[9] Espinel-Ingroff A, Chakrabarti A, Chowdhary A, Cordoba S, Dannaoui E, Dufresne P, et al. Multicenter evaluation of MIC distributions for epidemiologic cutoff value definition to detect amphotericin B, posaconazole, and itraconazole resistance among the most clinically relevant species of Mucorales. Antimicrob Agents Chemother. 2015;59(3):1745–50. Epub 2015/01/15. doi: 10.1128/AAC.04435-14 ; PubMed Central PMCID: PMC4325796. 25583714 PMC4325796

[10] Cornely OA, Alastruey-Izquierdo A, Arenz D, Chen SCA, Dannaoui E, Hochhegger B, et al. Global guideline for the diagnosis and management of mucormycosis: an initiative of the European Confederation of Medical Mycology in cooperation with the Mycoses Study Group Education and Research Consortium. Lancet Infect Dis. 2019;19(12):e405–e21. Epub 20191105. doi: 10.1016/S1473-3099(19)30312-3 ; PubMed Central PMCID: PMC8559573. 31699664 PMC8559573

[11] Lanternier F, Poiree S, Elie C, Garcia-Hermoso D, Bakouboula P, Sitbon K, et al. Prospective pilot study of high-dose (10 mg/kg/day) liposomal amphotericin B (L-AMB) for the initial treatment of mucormycosis. J Antimicrob Chemother. 2015;70(11):3116–3123. doi: 10.1093/jac/dkv236 26316385

[12] Rudramurthy SM, Hoenigl M, Meis JF, Cornely OA, Muthu V, Gangneux JP, et al. ECMM/ISHAM recommendations for clinical management of COVID-19 associated mucormycosis in low- and middle-income countries. Mycoses. 2021;64(9):1028–37. Epub 20210726. doi: 10.1111/myc.13335 ; PubMed Central PMCID: PMC8447004. 34133816 PMC8447004

[13] Marty FM, Ostrosky-Zeichner L, Cornely OA, Mullane KM, Perfect JR, Thompson GR, 3rd, et al. Isavuconazole treatment for mucormycosis: a single-arm open-label trial and case-control analysis. Lancet Infect Dis. 2016;16(7):828–37. Epub 2016/03/13. doi: 10.1016/S1473-3099(16)00071-2 . 26969258

[14] Nagappan V, Deresinski S. Reviews of anti-infective agents: posaconazole: a broad-spectrum triazole antifungal agent. Clin Infect Dis. 2007;45(12):1610–7. Epub 2008/01/15. doi: 10.1086/523576 . 18190324

[15] Dannaoui E, Meletiadis J, Mouton JW, Meis JF, Verweij PE. In vitro susceptibilities of zygomycetes to conventional and new antifungals. J Antimicrob Chemother. 2003;51(1):45–52. Epub 2002/12/21. doi: 10.1093/jac/dkg020 . 12493786

[16] Greenberg RN, Mullane K, J-AHv B, Raad I, Abzug MJ, Anstead G, et al. Posaconazole as Salvage Therapy for Zygomycosis. Antimicrob Agents Chemother. 2006;50(1):126–133. doi: 10.1128/AAC.50.1.126-133.2006 16377677 PMC1346806

[17] Badali H, Cañete-Gibas C, McCarthy D, Patterson H, Sanders C, David MP, et al. Epidemiology and Antifungal Susceptibilities of Mucoralean Fungi in Clinical Samples from the United States. J Clin Microbiol. 2021;59(9):e0123021. Epub 20210818. doi: 10.1128/JCM.01230-21 ; PubMed Central PMCID: PMC8373021. 34232068 PMC8373021

[18] Salas V, Pastor FJ, Calvo E, Alvarez E, Sutton DA, Mayayo E, et al. In vitro and in vivo activities of posaconazole and amphotericin B in a murine invasive infection by Mucor circinelloides: poor efficacy of posaconazole. Antimicrob Agents Chemother. 2012;56(5):2246–50. Epub 20120130. doi: 10.1128/AAC.05956-11 ; PubMed Central PMCID: PMC3346623. 22290952 PMC3346623

[19] Graves B, Morrissey CO, Wei A, Coutsouvelis J, Ellis S, Pham A, et al. Isavuconazole as salvage therapy for mucormycosis. Medical Mycology Case Reports. 2016;11:36–9. doi: 10.1016/j.mmcr.2016.03.002 27158585 PMC4845387

[20] Roilides E, Antachopoulos C. Isavuconazole: an azole active against mucormycosis. Lancet Infect Dis. 2016;16(7):761–2. Epub 20160309. doi: 10.1016/S1473-3099(16)00127-4 . 26969257

[21] Schwarz P, Cornely OA, Dannaoui E. Antifungal combinations in Mucorales: A microbiological perspective. Mycoses. 2019;62(9):746–60. doi: 10.1111/myc.12909 30830980

[22] Reed C, Bryant R, Ibrahim AS, Edwards J Jr., Filler SG, Goldberg R, et al. Combination polyene-caspofungin treatment of rhino-orbital-cerebral mucormycosis. Clinical infectious diseases: an official publication of the Infectious Diseases Society of America. 2008;47(3):364–71. Epub 2008/06/19. doi: 10.1086/589857 ; PubMed Central PMCID: PMC2793535. 18558882 PMC2793535

[23] Gebremariam T, Gu Y, Singh S, Kitt TM, Ibrahim AS. Combination treatment of liposomal amphotericin B and isavuconazole is synergistic in treating experimental mucormycosis. J Antimicrob Chemother. 2021;76(10):2636–2639. doi: 10.1093/jac/dkab233 34263306 PMC8446914

[24] Cornely OA, Arikan-Akdagli S, Dannaoui E, Groll AH, Lagrou K, Chakrabarti A, et al. ESCMID and ECMM joint clinical guidelines for the diagnosis and management of mucormycosis 2013. Clin Microbiol Infect. 2014;20 Suppl 3:5–26. Epub 2014/02/01. doi: 10.1111/1469-0691.12371 . 24479848

[25] Spellberg B, Ibrahim A, Roilides E, Lewis RE, Lortholary O, Petrikkos G, et al. Combination therapy for mucormycosis: why, what, and how? Clin Infect Dis. 2012;54 Suppl 1(Suppl 1):S73-8. Epub 2012/01/25. doi: 10.1093/cid/cir885 ; PubMed Central PMCID: PMC4542574. 22247449 PMC4542574

[26] Vironneau P, Kania R, Morizot G, Elie C, Garcia-Hermoso D, Herman P, et al. Local control of rhino-orbito-cerebral mucormycosis dramatically impacts survival. Clin Microbiol Infect. 2014;20(5):O336–9. Epub 20131106. doi: 10.1111/1469-0691.12408 . 24118291

[27] Ma LJ, Ibrahim AS, Skory C, Grabherr MG, Burger G, Butler M, et al. Genomic analysis of the basal lineage fungus Rhizopus oryzae reveals a whole-genome duplication. PLoS Genet. 2009;5(7):e1000549. Epub 20090703. doi: 10.1371/journal.pgen.1000549 ; PubMed Central PMCID: PMC2699053. 19578406 PMC2699053

[28] Gebremariam T, Alkhazraji S, Lin L, Wiederhold NP, Garvey EP, Hoekstra WJ, et al. Prophylactic Treatment with VT-1161 Protects Immunosuppressed Mice from Rhizopus arrhizus var. arrhizus Infection. Antimicrob Agents Chemother. 2017;61(9). Epub 20170824. doi: 10.1128/AAC.00390-17 ; PubMed Central PMCID: PMC5571349. 28652241 PMC5571349

[29] Gebremariam T, Alkhazraji S, Alqarihi A, Wiederhold NP, Shaw KJ, Patterson TF, et al. Fosmanogepix (APX001) Is Effective in the Treatment of Pulmonary Murine Mucormycosis Due to Rhizopus arrhizus. Antimicrob Agents Chemother. 2020;64(6). Epub 20200521. doi: 10.1128/AAC.00178-20 ; PubMed Central PMCID: PMC7269494. 32205345 PMC7269494

[30] Gebremariam T, Gu Y, Alkhazraji S, Youssef E, Shaw KJ, Ibrahim AS. The Combination Treatment of Fosmanogepix and Liposomal Amphotericin B Is Superior to Monotherapy in Treating Experimental Invasive Mold Infections. Antimicrob Agents Chemother. 2022;66(7):e0038022. Epub 20220607. doi: 10.1128/aac.00380-22 ; PubMed Central PMCID: PMC9295579. 35670592 PMC9295579

[31] Hoenigl M, Sprute R, Egger M, Arastehfar A, Cornely OA, Krause R, et al. The Antifungal Pipeline: Fosmanogepix, Ibrexafungerp, Olorofim, Opelconazole, and Rezafungin. Drugs. 2021;81(15):1703–29. Epub 20211009. doi: 10.1007/s40265-021-01611-0 ; PubMed Central PMCID: PMC8501344. 34626339 PMC8501344

[32] Vellanki S, Billmyre RB, Lorenzen A, Campbell M, Turner B, Huh EY, et al. A Novel Resistance Pathway for Calcineurin Inhibitors in the Human-Pathogenic Mucorales Mucor circinelloides. MBio. 2020;11(1):e02949–19. doi: 10.1128/mBio.02949-19 . 31992620 PMC6989107

[33] Park H-S, Lee SC, Cardenas ME, Heitman J. Calcium-Calmodulin-Calcineurin Signaling: A Globally Conserved Virulence Cascade in Eukaryotic Microbial Pathogens. Cell Host Microbe. 2019;26(4):453–62. doi: 10.1016/j.chom.2019.08.004 31600499 PMC6788756

